# Expanding Genotype/Phenotype Correlation in 2p11.2-p12 Microdeletion Syndrome

**DOI:** 10.3390/genes14122222

**Published:** 2023-12-16

**Authors:** Alessandra Ferrario, Nijas Aliu, Claudine Rieubland, Sébastian Vuilleumier, Hilary M. Grabe, Pascal Escher

**Affiliations:** 1 Department of Ophthalmology, Inselspital, Bern University Hospital, University of Bern, 3010 Bern, Switzerland; alessandra.ferrario@students.unibe.ch (A.F.); sebastian.vuilleumier@insel.ch (S.V.); hilary.grabe@insel.ch (H.M.G.); 2 Department of Human Genetics, Inselspital, Bern University Hospital, University of Bern, 3010 Bern, Switzerland; nijas.aliu@insel.ch (N.A.);; 3 Department of BioMedical Research, University of Bern, 3010 Bern, Switzerland

**Keywords:** microdeletion, chromosome 2, neurodevelopmental disorder, facial malformations, congenital ear anomalies, *POLR1A*, *REEP1*, *ELMOD3*, *FOXI3*

## Abstract

Chromosomal abnormalities on the short arm of chromosome 2 in the region p11.2 have been associated with developmental delay, intellectual disability, facial anomalies, abnormal ears, skeletal and genital malformations. Here we describe a patient with a de novo interstitial heterozygous microdeletion on the short arm of chromosome 2 in the region p11.2-p12. He presents with facial dysmorphism characterized by a broad and low root of the nose and low-set protruding ears. Clinical examinations during follow-up visits revealed congenital pendular nystagmus, decreased visual acuity and psychomotor development disorder including intellectual disability. The heterozygous 5 Mb-microdeletion was characterized by an array CGH (Comparative Genomic Hybridization) analysis. In the past two decades, nine patients with microdeletions in this region have been identified by array CGH analysis and were reported in the literature. All these patients show psychomotor development disorder and outer and/or inner ear anomalies. In addition, most of the patients have mild to severe intellectual disability and show facial malformations. We reviewed the literature on PubMed and OMIM using the gene/loci names as search terms in an attempt to identify correlations between genes located within the heterozygous microdeletion and the clinical phenotype of the patient, in order to define a recognizable phenotype for the 2p11.2p12 microdeletion syndrome. We discuss additional symptoms that are not systematically present in all patients and contribute to a heterogeneous clinical presentation of this microdeletion syndrome.

## 1. Introduction

Chromosomal abnormalities often affect multiple organ systems [1]. These are rare events, but the more individuals with a similar phenotype are described in the literature, the better a genetic disease can be characterized by a recognizable phenotype, despite heterogeneous clinical presentation and variable penetrance [2]. So far, nine patients with an interstitial heterozygous microdeletion on the short arm of chromosome 2 in the region p11.2-p12 have been identified by array CGH (Comparative Genomic Hybridization) analysis [3,4,5,6,7,8,9,10]. The first reported case with an interstitial microdeletion located in 2p11.2-p12 described a 5-year-old boy with microcephaly, a high forehead, broad high nasal bridge, ear anomalies, feet and digital anomalies, delayed psychomotor development, intellectual disability and speech delay [3]. A second patient was described shortly thereafter as having the following common characteristics: a similar facial dysmorphism, delayed motor development, intellectual disability and speech delay. Additionally, the patient was affected by ataxia and a congenital vesicoureteral reflux [4]. In a third case with a similar chromosomal microdeletion, ear anomalies, a high forehead, broad high nasal bridge, delayed psychomotor development and delayed speech progression were reported, and, for the first time, hearing impairment [5]. Four additional patients with overlapping interstitial microdeletions in 2p11.2-p12 all had ear anomalies [6,7,8]. Three patients also showed facial dysmorphism and were diagnosed with psychomotor development delay [6,8]. Because the fourth patient was described at the age of 4 months, the development progress could not be evaluated properly [7]. This young patient was, however, the first one not showing facial malformations, but showed left aural atresia and ipsilateral internal carotid artery agenesis [7]. An additional patient also presented with ear anomalies, facial dysmorphism, speech delay and delayed psychomotor development [10]. This patient is the only one described as an adult, at the age of 35 years, when she developed an atypical early-onset parkinsonism [10]. Finally, a patient with a homozygous 25 kb microdeletion spanning the *ELMOD3*, *CAPG* and *SH2D6* genes was reported [9].

Here we describe another patient with a de novo interstitial chromosomal 2p11.2-p12 heterozygous microdeletion characterized by array CGH analysis. To understand the function of the genes affected by the microdeletion in our patient, the literature on PubMed and OMIM was reviewed using each gene name as a search term. We discuss this literature review in light of the clinical symptoms present in patients harboring 2p11.2-p12 microdeletions and in an attempt to establish genotype–phenotype correlations.

## 2. Materials and Methods

### 2.1. Study Design

**Figure 1:**
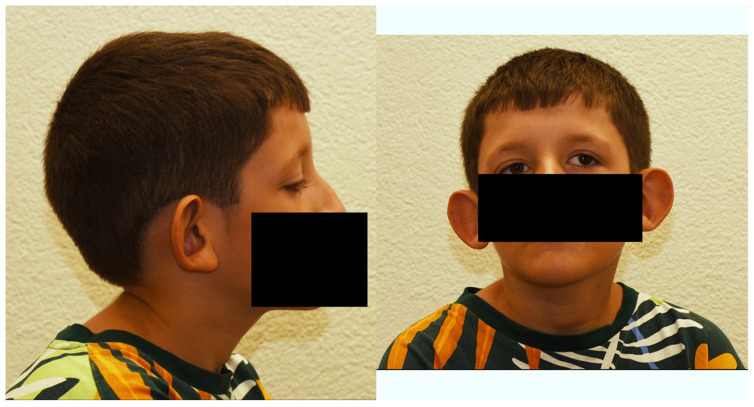
Facial images of the described patient at the age of 12 years. The side view (**left** panel) shows the low-set protruding ears. The front view (**right** panel) shows the broad and low root of the nose, the sloping eyelid axis and ocular torticollis.

All aspects of this retrospective study adhered to the tenets of the Declaration of Helsinki. The patient was recruited from the Inselspital, Bern University Hospital, Bern, Switzerland. The study was conducted in accordance with the Swiss Human Research Act and the International Council for Harmonisation of Technical Requirements for Registration of Pharmaceuticals for Human Use (ICH) guidelines of Good Clinical Practice (GCP). Written informed consent was given by the parents of the patient for the scientific use of clinical and molecular genetic data. We performed a medical record review. The mother of the patient provided further information. The photographs of Figure 1 were prepared in close collaboration with the mother of the proband. An additional written consent for the use of these photographs was obtained from the parents. Additional patients discussed in this article were all previously published.

### 2.2. Molecular Genetic Analysis

For standard karyotyping, peripheral blood lymphocytes were extracted from heparin blood and cultivated. GTG (G-bands after trypsin and Giemsa) banding was performed at an ISCN (International System for Human Cytogenetic Nomenclature) quality of 550 bands on metaphase chromosomes of 10 cells. Parental DNA was further analyzed by fluorescence in situ hybridization (FISH) using whole chromosome painting for chromosome 2 (WCP-2; Q-Biogene; Heidelberg, Germany). Molecular karyotyping was performed by array CGH analysis using the 4x135k PerkinElmer CGX-Array (PerkinElmer, Waltham, MA, USA). DNA was isolated from peripheral blood lymphocytes using the Qiagen Maxi-Kit (Qiagen, Hilden, Germany). Bioinformatic analysis was based on the human reference genome hg18, build 36, and performed with Genoglyphix 3.0 software (PerkinElmer). Additionally, the chromosomal region 15q11 was analyzed by MLPA (multiplex ligation dependent Probe Amplification) for deletions, duplications and methylation using ME028-B2 test (MRC Holland, Amsterdam, The Netherlands).

### 2.3. Literature Search

OMIM (Online Mendelian Inheritance in Man) and PubMed databases were, respectively, accessed at https://www.omim.org/ and https://pubmed.ncbi.nlm.nih.gov/ from 10 August 2021 to 01 May 2023. We used ‘official HGNC (Human Genome Nomenclature Committee) gene symbol’ as the search term in OMIM. On PubMed we used ‘official HGNC gene symbol AND eye’ as search term.

## 3. Results

### 3.1. Clinical Case Report

We describe the first child of a healthy, non-consanguineous Swiss–Kosovan couple. The patient is presently 12 years old (Figure 1). He has a 4-year-old younger, unaffected sister. The patient had his initial visits in the Inselspital, Bern University Hospital, Bern, Switzerland with continued periodic follow-up examinations there.

The mother, then aged 22 years, gave birth after an uncomplicated pregnancy with vaginal delivery at term. The boy had a birth weight of 2540 g (1–2th centile), a head circumference of 32 cm (<1th centile) and a birth length of 47 cm (1–2th centile). Apgar score was 8, 9 and 10 at 1, 5 and 10 min respectively. He was born with a bilateral syndactyly of toes II/III and facial dysmorphism including a flat midface, broad and low nasal root, narrow lips, low-set protruding ears and downslanting palpebral fissures. After birth, the first clinical concerns were episodes of cyanosis during feeding and the inability to drink due to hypotension of the oropharyngeal muscles. Later, a muscular hypotension of shoulder and torso muscles was diagnosed and resulted in delayed motor development (sitting without support at 11 months, walking alone at 22 months) and difficulties in coordination and fine motor skills. Further assessments revealed delayed cognitive skills and a development quotient of 64 to 86 over time. Speech progression was normal under speech therapy. Hearing assessment and brain MRI were normal.

An ophthalmological examination at the age of 4 months showed a horizontal pendular nystagmus to the left and high-degree visual impairment. Visual impairment was thought to be secondary to possible optic atrophy, but given the patient’s age and nystagmus a conclusive examination of the optic nerve was not possible at this time. Ophthalmological follow-up examinations at the age of 4, 6 and 8 years showed the persistence of the horizontal pendular nystagmus. Ocular alignment was orthophor. In addition, ocular torticollis (left-hand turn of 30° and tilt to the right) and astigmatism were evident. At the most recent follow-up visit at the age of 12 years, the pendular nystagmus and the ocular torticollis had remained stable. The anterior and posterior segments were normal, including the optic nerve head. Importantly, the distance visual acuity had markedly improved (Table 1).

The patient has a friendly and helpful disposition and good social skills without behavioral disturbances. He profits from support in a special school and shows high interest in social activities, playing with his healthy younger sister and in making intellectual progress.

### 3.2. Genetic Analysis

**Figure 2:**
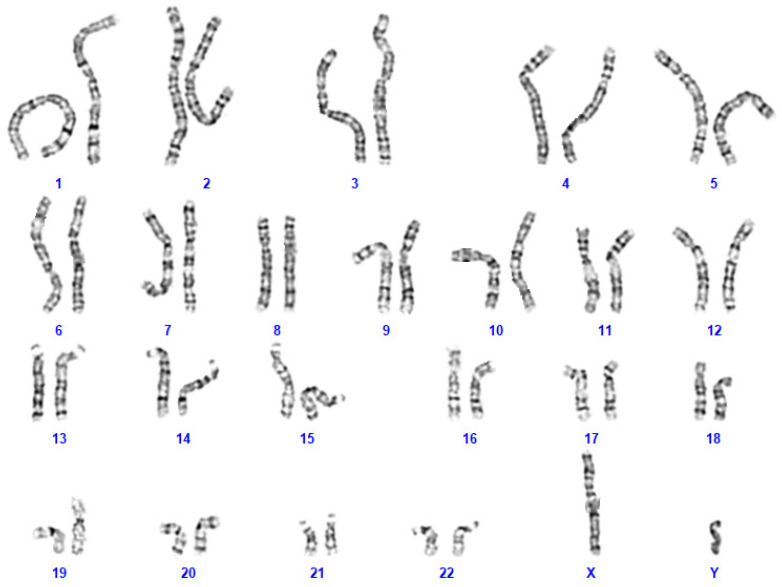
Karyotyping of the proband by standard GTG banding identified a 46,XY,del(2)(p11.2p11.2) karyotype.

Upon genetic counselling of the parents, genetic analyses were initiated at the age of 2 years. Karyotyping by GTG banding identified an interstitial chromosomal 2p11.2 heterozygous microdeletion (Figure 2). Karyotyping by GTG banding of the parents did not reveal any chromosomal abnormalities. A detailed FISH analysis of parental chromosomes 2 did not identify any chromosomal abnormalities neither. Taken together, these chromosomal analyses indicated that the heterozygous microdeletion had appeared de novo in the patient. Angelman Syndrome was excluded by detailed analysis of the patient’s chromosomal region 15q11.

**Figure 3:**
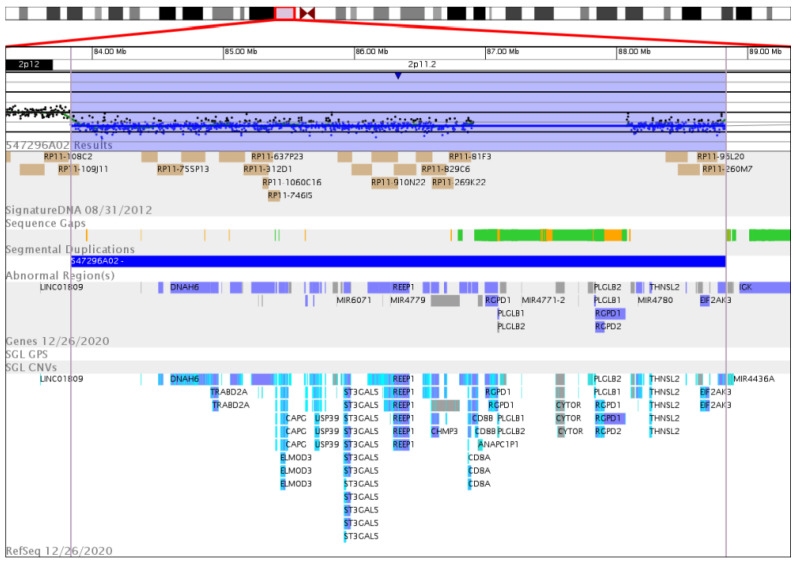
Array CGH analysis showing the heterozygous interstitial microdeletion arr[hg18] 2p11.2(83,836,581_88,835,028)x1 in detail. The 2p11.2 chromosomal region (top row, red box) is enlarged to show in detail (from top to down): probe array signals (black dots) with heterozygously deleted region (shaded in blue); sequence gaps (green, orange); deleted genes and loci (gray, blue, light blue). This image has been extracted from www.genoglyphix.com (PerkinEmer).

The array CGH analysis then revealed an interstitial heterozygous microdeletion of 5 Mb on the short arm of chromosome 2 at p11.2. The deletion located close to the centromere spanned from position 83,836,581 to position 88,835,028 according to UCSC genome browser assembly GRCh36/hg18: arr[hg18] 2p11.2(83,836,581_88,835,028)x1 (Figure 3).

This region harbors 46 protein-coding genes, three pseudogenes, eight microRNAs, five other RNAs (nucleolar, non-coding, anti-sense) and five undescribed loci (Table 2).

### 3.3. Literature Search

To identify disease-associated genes within the deleted region, we searched the literature on the OMIM (Online Mendelian Inheritance in Man) and PubMed databases by using the ‘gene name’ as the search term. To search for eye-specific data pertinent to decreased visual acuity and nystagmus, an additional search was performed on PubMed using the search term ‘gene name AND eye’. We accessed OMIM and PubMed from 10.08.2021 to 01.05.2023.

According to OMIM, 12 out of the 46 protein-coding genes have been associated with human disease (*SUCLG1*, *ELMOD3*, *GGCX*, *SFTPB*, *ST3GAL5*, *POLR1A*, *PTCD3*, *REEP1*, *CD8A*, *FOXI3*, *EIF2AK3*, *RP1A*) (Table 2). For eight genes (*SUCLG1*, *GGCX*, *SFTPB*, *ST3GAL5*, *PTCD3*, *CD8A*, *EIF2AK3*, *RP1A*), autosomal recessive inheritance has been established. These genes are therefore unlikely to contribute to the clinical phenotype. Both recessive and dominant inheritance has been associated with *ELMOD3* and *FOXI3*. For *POLR1A* and *REEP1*, autosomal dominant inheritance with loss-of-function as a disease mechanism has been reported.

## 4. Discussion

To better define the symptoms from the clinical findings present in our patient, we reviewed all above-mentioned patients with interstitial chromosomal 2p11.2-p12 microdeletions characterized by array CGH analysis [3,4,5,6,7,8,9,10]. We report the published clinical symptoms and the exact genomic localization for all patients (Table 3).

**Figure 4:**
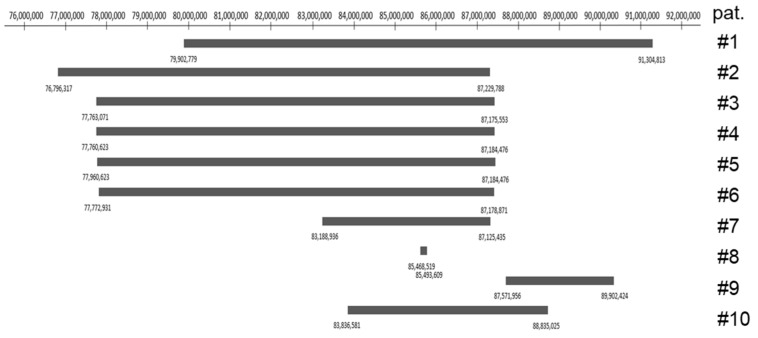
Overview of the deleted regions in the previously reported patients and the patient described in this article. The numbers mark the position on chromosome 2 at the band 2p11.2p12 according to UCSC genome browser assembly GRCh36/hg18. On the right side, patients are numbered according to Table 3.

To better visualize the microdeletions of the 10 patients and overlapping deleted regions, we generated a synoptic view of the available array CGH data (Figure 4).

Diseases with autosomal dominant inheritance have been linked to four genes located on the heterozygously deleted chromosomal region: *POLR1A*, *ELMOD*, *FOXI3* and *REEP1*.

All 10 patients mentioned above show abnormalities in the formation of the outer and/or the inner ear. With the exception of patients 8 and 9, the deleted genomic regions contain in all patients the *POLR1A* gene coding for the 194-kD α subunit of the RNA polymerase 1 (OMIM #616404). Pathogenic heterozygous variants in *POLR1A* are associated with acrofacial dysostosis, Cincinnati type, the symptoms of which include large and low-set ears, downslanting palpebral fissures, an underdeveloped midface, micrognathia, decreased head circumference and short stature [11,12]. Haploinsufficiency in *POLR1A,* as present in our patient with a heterozygous 2p11.2 microdeletion, may therefore be the genetic cause of the large and low-set ears (Figure 1), and contribute to the observed downslanting palpebral fissures and facial dysmorphism with flat midface.

In patient 8, the small 25 kb homozygous deletion affects only three genes, *ELMOD3*, *CAPG* and *SH2D6* [9]. Based on the reported autosomal recessive inheritance in a consanguineous Pakistani family affected with non-syndromic deafness (DFNB88; OMIM #615429) [13], hearing loss was attributed to the homozygous deletion of *ELMOD3* in this patient. Because hearing loss is not reported in the patients with a heterozygous deletion of *ELMOD3*, except for patient 3, the disease mechanism in a three-generation Chinese pedigree reported with *ELMOD3*-linked autosomal dominant non-syndromic deafness (DNFA81; OMIM #619500) may involve dominant-negative effects rather than haploinsufficiency [14,15,16]. Large ears are also reported in patient 8 [9], and a contribution to the external ear phenotype of one of three genes *ELMOD3*, *CAPG* and *SH2D6* cannot be excluded.

In contrast to the other patients with reported large ears, microtia is reported in patient 9 [7]. Consistently, *POLR1A* is not deleted in this patient. The heterozygous microdeletion harbors the *FOXI3* gene, coding for the transcription factor forkhead box I3, involved in the development of teeth, hair and the inner ear (OMIM #612351) (Figure 4). Recently, heterozygous pathogenic variants in *FOXI3* have been associated with craniofacial microsomia, also known as Goldenhar syndrome, whose cardinal symptoms are microtia and craniofacial microsomia [17,18]. Our patient 10 is the only one among the reported patients in which both *POLR1A* and *FOXI3* are deleted. Based on the clinical symptoms of our patient with large and low-set ears (Figure 1), one might hypothesize that haploinsufficiency of *POLR1A* overrides that of *FOXI3* during ear development. Additionally, haploinsufficiency is not the sole disease mechanism in *FOXI3*-linked microtia, as recessive inheritance has been demonstrated in a consanguineous Pakistani family [17].

*REEP1* encodes the receptor expression-enhancing protein 1. Heterozygous pathogenic deletions, frameshift, splice-site, missense and 3′-UTR variants cause hereditary spastic paraplegia type 31 (SPG31) [19] and a splice-site variant hereditary motor neuronopathy 12 (HMND12; OMIM 614751) [20]. All these variants may eventually lead to haploinsufficiency [19]. SPG31 is a pure hereditary spastic paraplegia leading to progressive spasticity of the lower limbs, with a rare complication of peripheral nerve involvement [21,22,23]. Initial symptoms in SPG31 patients appear in a bimodal pattern, before the age of 20 years or after the age of 30 years, and penetrance is incomplete even at an advanced age [21]. Strikingly, patient 7 is the only adult patient reported so far and her clinical symptoms of an atypical early onset parkinsonism include dystonia and lower limb spasticity [10]. Based on this report, neurological follow-up examinations of our patient will include nerve conduction studies.

Only our patient and patient 7 [10] have ocular anomalies, such as strabismus, amblyopia, decreased visual acuity and nystagmus (Table 3). These two patients share the most similar interstitial microdeletion (Figure 4). The improvement in distance visual acuity observed in our patient is remarkable. The medical history of patient 7 also mentions amblyopia, but no decrease in visual acuity is reported at age 35 years [10], suggesting a similar positive development in visual acuity for this patient.

Shared heterozygous deleted genes for which expression in the eye has been reported include *TRABD2A*, *TMSB10*, *RETSAT*, *MAT2A*, *GGCX*, *ST3GAL5*, *PTCD3*, *RPIA. TRABD2A* encodes the 505-aa TIKI1 protein, a negative regulator of Wnt signaling during embryonic development of the eye [24]. *TMSB10* encodes thymosin β-10 expressed during early human retinogenesis [25]. *RETSAT* encodes the retinol saturase catalyzing the saturation of all-trans-retinol to dihydroretinoid metabolites [26]. This process is not involved in the vitamin A cycle of the visual system, but in the absence of retinol saturase, mice are prone to adiposity [26]. *MAT2A* encodes the catalytic part of the methionine adenosyltransferase 2, catalyzing the synthesis of the methyl donator S-adenosylmethionine (SAM). Its expression is upregulated during the acute phase after brain, retinal and spinal cord injury [27]. No specific disease has been associated so far with defects in *TRABD2A, TMSB10, RETSAT* and *MAT2A*. Homozygous or compound heterozygous pathogenic variants in *GGCX* are associated with a pseudoxanthoma elasticum-like disorder with multiple coagulation factor deficiency (OMIM #610842) and the combined deficiency of the vitamin K-dependent clotting factor (OMIM #277450). A single patient is described with motor ataxia of the eye [28], but this pseudoxanthoma elasticum-like disorder also affects the optic nerve and the retina [29]. Given that exclusively autosomal recessive inheritance has been reported, a heterozygous deletion of *GGCX* is unlikely to contribute to the clinical phenotype in these two patients. *ST3GAL5* encodes the ST3 β-galactoside α-2,3-silyltransferase 5. This enzyme catalyzes the first step of the synthesis of several different gangliosides (OMIM #604402). Homozygous or compound heterozygous pathogenic variants in *ST3GAL5* cause the salt and pepper developmental regression syndrome (MIM #609056). *PTCD3* encodes the pentatricopeptide repeat domain-containing protein 3, which is essential for translation in mitochondria (MIM #614918). Homozygous and compound heterozygous pathogenic variants in *PTCD3* cause the combined oxidative phosphorylation deficiency 51 (COXPD51), for which symptoms include respiratory insufficiency, nystagmus, delayed psychomotor development, brain lesions, bilateral hearing loss, and optic atrophy (OMIM #619057) [30]. *RPIA* encodes ribose 5-phosphate isomerase A, an enzyme of the pentose phosphate pathway (OMIM #180430). Homozygous and compound heterozygous pathogenic variants in *RPIA* cause ribose 5-phosphate isomerase deficiency (RPIAD; OMIM #608611). In a cohort of four patients, development delay, speech delay and alterations in cerebral white matter were always present, and ocular phenotypes included optic atrophy in one patient, exotropia and retinitis pigmentosa in the others [31]. Again, only autosomal recessive inheritance has been reported for *ST3GAL5, PTCD3* and *RPIA.*

Our patient suffers from muscular hypotension of shoulder and torso muscles. Patient 4 is described to have a hypotonic habitus, which is absent in patient 5, despite them harboring an identical microdeletion to patient 4 [6]. This suggests that either the microdeletion is not responsible for this phenotype or that only compound heterozygous mutations in the overlapping region are causative. Our literature search identified no correlation between genomic defects in the mentioned region and muscular hypotension.

Of note, only in patients 1 and 9 is the malformation of vessels reported [3,7]. The microdeletion in these two patients spans further from the centromere compared to the other deletions listed in Figure 4. This could suggest that deletions between 88′835,025 and 91,304,813 have an impact on the formation of vessels. A literature review of the affected genes could help to support this statement.

In conclusion, the heterozygous deletion of *POLR1A* may contribute most to the clinical phenotype in interstitial chromosomal 2p11.2p12 microdeletions, causing large and low-set ears, downslanting palpebral fissures and facial dysmorphism with flat midface. Importantly, follow-up examinations of patients with heterozygous *REEP1* deletion should include specific neurological tests to assess spasticity of the lower limbs and peripheral nerve involvement, and this should carry on through adulthood.

Fortunately, the patient newly reported here has shown a satisfactory visual development. The collaboration between the ophthalmologists and neuro-pediatricians at our hospital and the special school addressing visual and intellectual support he needs continues on a regular schedule. This is part of the multidisciplinary care typically needed for patients with chromosomal abnormalities. Genetic counselling and analysis is a cornerstone of this comprehensive care and could be initiated early in life, thanks to supportive parents.

## Figures and Tables

**Table 1:** Distant and near visual acuity of binocular vision (OU), monocular vision of the left eye (OS) and monocular vision of the right eye (OD) at age 4, 6, 8 and 12 years.

Age (years)	4	6	8	12
Distance visual acuity	OU: 0.63	OD: 0.16 OS: 0.2	OD: 0.5 OS: 0.63	OU: 1.0
Near visual acuity	OU: 0.5	OD: 0.16	OD: 0.63 OS: 0.25	OD: 0.4 OS: 0.5

**Table 2:** List of loci identified within the interstitial chromosomal 2p11.2 microdeletion. The region is described from 5′ to 3′. Gene: official HGNC gene symbols; gene product: full gene or protein name; inheritance: autosomal dominant (AD), autosomal recessive (AR); disease: gene-associated human diseases; OMIM (Online Mendelian Inheritance in Man) numbers are listed. In bold, genes for which autosomal dominant inheritance has been reported.

Gene	Gene Product	Inheritance	Disease	OMIM
*FUNDC2P2*	FUN14 domain containing 2 pseudogene 2			
*SUCLG1*	Succinate-CoA Ligase, GDP/ADP-forming, α subunit	AR	Mitochondrial DNA depletion syndrome 9	245400
*DNAH6*	Dynein, axonemal, heavy chain 6			
*TRABD2A*	TRAB domain-containing protein 2a			
*TMSB10*	Thymosin, β-10			
*KCMF1*	Potassium channel modulatory factor 1			
*LINC01964*	Long intergenic non-protein coding RNA 1964			
*TCF7L1-IT1*	TCF7L1 intronic transcript 1			
*TCF7L1*	Transcription factor 7-like 1			
*LOC102724579*				
*TGOLN2*	Trans-Golgi network protein 2			
*RETSAT*	Retinol saturase			
***ELMOD3***	**ELMO/CED12 domain-containing protein 3**	**AD** AR	**Deafness 81** Deafness 88	**619500** 615429
*CAPG*	Capping protein, gelsolin-like			
*SH2D6*	SH2 domain containing 6			
*PARTICL*	Promoter of MAT2 AS radiation-induced circulating lncRNA			
*MAT2A*	Methionine adenosyltransferase 2A			
*GGCX*	γ-glutamyl carboxylase	AR	Combined deficiency of Vitamin K-dependent clotting factors Pseudoxanthoma elasticum-like disorder withmultiple coagulation factor deficiency	277450 610842
*VAMP8*	Vesicle-associated membrane protein 8			
*VAMP5*	Vesicle-associated membrane protein 5			
*RNF181*	Ring finger protein 181			
*TMEM150A*	Transmembrane protein 150a			
*USP39*	Ubiquitin-specific protease 39			
*C2ORF68*	Chromosome 2 open reading frame 68			
*SFTPB*	Surfactant protein B	AR	Pulmonary surfactant metabolism dysfunction 1	265120
*GNLY*	Granulysin			
*ATOH8*	Atonal-homolog 8			
*MIR6071*	MicroRNA 6071			
*LOC284950*				
*ST3GAL5*	ST3 β-galactoside α-2,3-sialyltransferase 5	AR	Salt and pepper developmental regression syndrome	609056
*ST3GAL5-AS1*	ST3GAL5 antisense RNA 1			
***POLR1A***	**RNA polymerase I subunit A**	**AD**	**Acrofacial dysostosis, Cincinnati type**	**616462**
*PTCD3*	Pentatricopeptide repeat domain 3	AR	Combined oxidative phosphorylation deficiency 51	619057
*SNORD94*	Small nucleolar RNA, C/D box 94			
*IMMT*	Inner membrane mitochondrial protein			
*MIR4779*	MicroRNA 4779			
*MRPL35*	Mitochondrial ribosomal protein L35			
***REEP1***	**Receptor expression-enhancing protein 1**	**AD** AR	**Spastic paraplegia 31** **Distal hereditary motor neuronopathy 12** Distal hereditary motor neuronopathy 6	**610250** **614751** 620011
*KDM3A*	Lysine-specific demethylase 3a			
*CHMP3*	Charged multivesicular body protein 3			
*RNF103*	Ring finger protein 103			
*RMND5A*	Required for meiotic nuclear division 5 homolog a			
*CD8A*	CD8 antigen, α polypeptide	AR	Familial CD8 deficiency	608957
*CD8B*	CD8 antigen, β polypeptide			
*ANAPC1P1*	ANAPC1 pseudogene 1			
*RGPD1*	RANBP2 like and GRIP domain containing 1			
*RGPD2*	RANBP2 like and GRIP domain containing 2			
*PLGLB2*	Plasminogen like B2			
*PLGLB1*	Plasminogen like B1			
*LOC285074*				
*MIR4771-2*	MicroRNA 4771-2			
*MIR4772-1*	MicroRNA 4771-1			
*CYTOR*	Cytoskeleton regulator RNA			
*MIR4435-1*	MicroRNA 4435-1			
*MIR4435-2*	MicroRNA 4435-2			
*KRCC1*	Lysine rich coiled-coil 1			
*FABP1*	Fatty acid binding protein 1			
*SMYD1*	SET and MYND domain-containing protein 1			
*MIR4780*	MicroRNA 4780			
*THNSL2*	Threonine synthase like 2			
***FOXI3***	**Forkhead box I3**	**AD/AR**	**Craniofacial microsomia 2**	**620444**
*TEX37*	Sperm microtubule inner protein 9			
*LOC101928371*				
*EIF2AK3*	Eukaryotic translation initiation factor 2 α kinase 3	AR	Wolcott–Rallison syndrome	226980
*LOC101928403*				
*RPIA*	Ribose 5-phosphate isomerase A	AR	Ribose 5-phosphate isomerase deficiency	608611
*ANKRD36BP2*	Ankyrin repeat domain 36B pseudogene 2			
*MIR4436A*	MicroRNA 4436a			

**Table 3:** Synoptic overview of microdeletion, patient data and clinical symptoms of all currently known patients with microdeletions in the genomic region 2p11.2p12. M: male; F: female; y: year; m: month; +: present; (+): subtle; -: absent; n.a.: not available.

Patient No.	1	2	3	4	5	6	7	8	9	10
Reference	Tzschach et al., 2009 [3]	Writzl et al., 2009 [4]	Rocca et al., 2012 [5]	Stevens et al., 2015 [6]	Stevens et al., 2015 [6]	Silipigni et al., 2015 [8]	Baviera-Munoz et al., 2021 [10]	Lahbib et al., 2019 [9]	Tassano et al., 2015 [7]	Present Case
Position in 2p	p11.2p12	p11.2p12	p11.2p12	p11.2p12	p11.2p12	p11.2p12	p11.2	p11.2	p11.2	p11.2
Deletion size	11.4 Mb	10.4 Mb	9.2 Mb	9.4 Mb	9.4 Mb	9.4 Mb	3.9 Mb	25.09 kb	2.4 Mb	5.0 Mb
Genomic Localization	79,902,77–91,304,813	76,796,317–87,229,788	77,763,071–87,175,553	77,760,623–87,184,476	77,760,623–87,184,476	77,772,931–87,178,871	83,188,936–87,125,435	85,468,519–85,493,609	87,571,956–89,902,424	83,836,581–88,835,025
Sex	F	M	F	M	M	F	F	M	F	M
Inheritance	de novo	de novo	de novo	de novo	de novo	de novo	de novo	parental, homozygous	paternal	de novo
Age evaluation	5 y	5 y 9 m	9 y	15 y 8 m	5 y 4 m	12 m	35 y	7 y	4 m	11 y 9 m
Microcephaly	+	-	-	-	-	-	-	-	-	-
High forehead	+	+	+	+	+	+	+	-	-	-
Broad high nasal bridge	+	+	+	+	+	+	(+)	-	-	+
Ear anomalies	+	+	+	+	+	+	+	+	+	+
Feet anomalies	+	+	-	+	-	-	-	-	-	+
Digital anomalies	+	+	-	-	-	-	-	-	-	-
Growth retardation	+	+	+	-	-	-	+	-	-	-
Speech delay	+	+	+	+	+	n.a.	-	+	n.a.	-
Delayed motor development	+	+	+	+	+	+	+	+	-	+
Hypertonia	-	+	-	hypotonia	-	-	-	hypotonia	-	hypotonia
Ataxia	-	+	+	-	-	-	-	-	-	-
Intellectual disability	+	mild	mild	moderate	borderline	mild	-	+	?	+
Happy disposition	+	+	-	+	+	+	?	-	?	+
Other symptoms	single umbilical artery	vesico-ureteral reflux	incomplete myelination white matter, hearing impairment	hyperreflexia of lower limbs, clumsy gait	hypermobile hands	bilateral choanal atresia, atrial septal defect	atypical early-onset parkinsonsim with dystonia and lower limb spasticity, scoliosis, strabismus, amblyopia	autism, hearing impairment, primary enuresis	congenital aural atresia, agenesis of internal carotid artery, velopharyngeal insufficiency, aberrant course of left facial nerve, microtia, hearing impairment	pendular horizontal nystagmus, visual acuity

## Data Availability

Data supporting reported results can be found on: https://omim.org, https://www.ncbi.nlm.nih.gov/pmc, and https://www.genoglyphix.com (accessed on 21 November 2022).
